# Updated guidelines on HIV post‐exposure prophylaxis: continued efforts towards increased accessibility

**DOI:** 10.1002/jia2.26393

**Published:** 2024-11-22

**Authors:** Lao‐Tzu Allan‐Blitz, Kenneth H. Mayer

**Affiliations:** ^1^ Division of Global Health Equity, Department of Medicine Brigham and Women's Hospital Boston Massachusetts USA; ^2^ Department of Medicine Harvard Medical School Boston Massachusetts USA; ^3^ The Fenway Institute of Fenway Health Boston Massachusetts USA; ^4^ Fenway Health Boston Massachusetts USA; ^5^ Division of Infectious Diseases, Department of Medicine Beth Israel Deaconess Medical Center Boston Massachusetts USA

**Keywords:** guidelines, HIV PEP, HIV post‐exposure prophylaxis, HIV prevention, policy, World Health Organization

## Abstract

**Introduction:**

HIV transmission is ongoing in both high‐ and low‐resource settings. Post‐exposure prophylaxis (PEP) remains an important tool in preventing HIV; however, PEP is significantly underutilized. The multitude of barriers to PEP implementation include low patient and provider awareness and acceptability, limited access to treatment and prevention services, and high rates of stigma. The World Health Organization (WHO) recently released updated guidance on the delivery of HIV PEP. This commentary aims to highlight the salient changes, evaluate how such recommendations can overcome the existing barriers to PEP implementation and discuss strategies needed to put the updated guidance into practice.

**Discussion:**

The 2024 WHO PEP guidelines continue a trend towards increasing access to PEP. Most notably, the WHO now provides strong recommendations that: (1) PEP be delivered in community settings (e.g. pharmacies, police stations and online platforms), and (2) PEP delivery and monitoring be done via task sharing involving non‐specialist health workers (e.g. pharmacists or community health workers). The guidelines also emphasize that the PEP encounter is an important educable moment whereby a transition to pre‐exposure prophylaxis among individuals at continued risk for HIV infection should be discussed. The decentralization of PEP delivery has the potential to overcome numerous barriers to PEP implementation, reduce time to initiation and support adherence with the 28‐day course. To translate the recommendations into delivery programmes, however, much more work is needed. Detailed templates can help overcome the heterogeneity of both the community settings in which PEP can now be provided and the populations (e.g. survivors of sexual assault, healthcare workers, sex workers, etc.) among whom PEP may be indicated. Training of the workforce will be essential, which should include, as emphasized by the WHO, training in trauma‐based care. Novel formulations of and delivery mechanisms for PEP are also emerging, and how such iterations can synergize with decentralized PEP delivery programmes remains to be seen.

**Conclusions:**

The updated WHO PEP guidelines make major strides towards increasing access to PEP. Realization of such aims will require ongoing evaluation and support given the heterogeneity in who benefits most from PEP.

## 1 INTRODUCTION

Despite significant progress made in the prevention and treatment of HIV, there were 1.3 million new HIV infections globally in 2022 [1]. Of the 30 million people with HIV who were aware of their status and engaged in care, 2.1 million (7%) were virologically unsuppressed [1]. An additional 5.5 million people were unaware of their status [1]. Thus, the potential for transmission remains a concern, and continues in both resource‐limited and resource‐rich contexts [2].

Pre‐exposure prophylaxis (PrEP) is highly efficacious [3, 4, 5, 6], and an essential component of the U.S. Centers for Disease Control and Prevention “Ending the HIV/AIDS epidemic” strategy [7]. However, significant disparities remain in access to and uptake of PrEP; in the United States, less than one‐third of people with an indication for PrEP have been prescribed the medications—with notable disparities in use among Black and Latinx individuals compared to White individuals [8]. Globally, the rollout of PrEP has also been slow due to challenges in uptake, adherence and persistence [9]. Furthermore, PrEP is not the optimal prevention strategy in many contexts, such as low‐frequency, high‐risk exposures.

Post‐exposure prophylaxis (PEP) thus continues to be another vital component of the HIV prevention strategy [10, 11]. However, driven by multiple concurrent and interrelated factors, PEP remains widely underutilized [12]. On 22 July 2024, the World Health Organization (WHO) revised its guidance on PEP delivery [13]. The updated guidelines include notable policy changes, but overall continue a trend that began with the 2014 WHO guidelines [10], aiming to increase the availability of and access to PEP as a preventive option (Table 1). This commentary aims to highlight the salient changes, evaluate how such recommendations can overcome the existing barriers to PEP implementation and describe the barriers that may exist to guideline adaptation.

**Table 1:** Key recommendations from the 2024 World Health Organization Guidelines on HIV postexposure prophylaxis [13]

Recommendation	Expected challenges	Potential solutions
Post‐exposure prophylaxis should be offered, and as early as possible, to individuals with suspected or known exposure to HIV, ideally within 24 hours but no later than 72 hours	Access to antiretroviral medications Low provider and patient awareness of PEP HIV‐related stigma, homo‐ and trans‐phobia	Assuring supply chain Community outreach programmes Campaigns to reduce stigma and combat stigmatizing policies Provider education programmes
An HIV post‐exposure prophylaxis regimen with two antiretroviral drugs is effective, but three drugs are preferred	Access to antiretroviral medications	Assuring supply chain
A 28‐day prescription of antiretroviral drugs should be provided for HIV post‐exposure prophylaxis following initial risk assessment	Low rates of completion Limited access to follow‐up services Low provider awareness	Community and patient outreach Implementation of adherence support strategies Provider education programmes
Enhanced adherence counselling is suggested for individuals initiating HIV post‐exposure prophylaxis	Nuanced and important differences in counselling for sub‐populations	Templates for providers in caring for different populations Training of providers in first‐line support for survivors of sexual violence
HIV post‐exposure prophylaxis should be delivered in community settings	Access to antiretroviral medications Sufficient infrastructure to support testing and medication delivery	Assuring supply chain Investment in local infrastructure
Task sharing should be employed to dispense, distribute and monitor HIV post‐exposure prophylaxis	Access to antiretroviral medications Heterogeneity in PEP delivery by non‐clinician personnel	Assuring supply chain Training of non‐clinician personnel

## 2 DISCUSSION

### 2.1 Existing barriers to PEP utilization and completion

In order to be efficacious, PEP must be administered within 72 hours of an exposure, creating a narrow window for intervention [14]. Any delays in presentation to care, identification of appropriate patients, medication prescription or availability of antiretroviral therapy will have sizable impacts on the utility of PEP. A recent review of reports published in the last 10 years found that 33% of more than 41,000 individuals eligible for PEP did not receive the medication; that report highlighted several structural, provider‐level and patient‐level factors associated with limited PEP initiation [12].

Structural factors include limited access to care [15, 16] and stockouts of essential medications [16] in many settings where PEP may be most useful. Additionally, some areas with a high prevalence of HIV lack human rights protections for individuals who engage in same‐sex relationships [17], which can create fears of persecution and limit both healthcare seeking and acceptability of PEP. Patient‐ and provider‐level factors that limit PEP use include low awareness of PEP as a preventive option [18, 19, 20], which may delay initiation, and low acceptability among different sub‐populations [21, 22, 23, 24]. Lack of provider familiarity with appropriate PEP regimens is another barrier [25], which is likely driven by low awareness, access and acceptability. Further, many providers may be uncomfortable or unfamiliar with eliciting a complete sexual history, which may make patients reluctant to discuss their exposures.

Even among those who initiate PEP, the proportion of individuals who complete the course is estimated to be nearly 57% overall: as high as 67% among men who have sex with men and as low as 40% among survivors of sexual assault [26]. Barriers to PEP completion include lack of access medication, inconvenience of follow‐up visits [27], lack of understanding of the need to complete the prescription and to be monitored for HIV seroconversion, social stigma and discrimination [28, 29], and, among survivors of sexual assault, specific trauma‐related factors [30]. Medication side effects may also limit PEP completion [29, 31, 32], although newer therapies are better tolerated [33].

### 2.2 Salient changes to international PEP guidelines

The 2014 WHO guidelines simplified eligibility assessment and PEP regimens, unifying the historically different guidelines by exposure (e.g. occupational, non‐occupational and sexual assault) [10, 34]. The 2024 guidelines now provide strong recommendations that: (1) PEP should be delivered in community settings (e.g. pharmacies, police stations and online platforms), and (2) PEP delivery and monitoring should be done via task sharing involving non‐specialist health workers (e.g. pharmacists or community health workers).

Those recommendations are based on a growing body of literature that supports the feasibility and effectiveness of PEP delivery in non‐healthcare settings [35, 36, 37, 38, 39, 40] and by individuals other than healthcare providers [35, 41–47]. Such recommendations also continue the trend of viewing PEP less as a healthcare‐centric biomedical intervention and more as one component of a larger community‐based and social effort to prevent HIV transmission. Strong support for community‐based PEP delivery has the potential to overcome the multitude of existing barriers. Bringing PEP into the community can facilitate easier and earlier access to care and normalize PEP as a tool, which may reduce stigma. Such changes may also increase awareness and potentially acceptability among both patients and providers.

Further, the recommendations also support improved adherence to the 28‐day course. Decentralization facilitates additional community‐based strategies to improve adherence, such as “adherence clubs,” and has become a key component of antiretroviral therapy adherence for people living with HIV [48]. Studies utilizing pharmacy‐based PEP delivery incorporated adherence reminders within the programme and demonstrated improved rates of PEP completion and follow‐up [41, 42]. Another study offering PEP in a variety of settings utilized physician‐driven text message reminders, and documented a similar improvement in PEP adherence [35]. The WHO also emphasizes adequate training in trauma‐informed care, in order to adequately support survivors of sexual assault who may face unique challenges in PEP adherence; one study highlighted the fact that PEP may be a daily reminder of the traumatic event for such individuals, limiting regimen completion [49].

In addition, the WHO further emphasizes that three‐drug regimens are preferred over two‐drug regimens, but that two‐drug regimens can be used if three‐drug regimens are unavailable. Further, based on available data, the most important consideration in the initiation of PEP initiation is the time since exposure [50, 51, 52, 53]; thus, the guidelines favour initiating whichever regimen is most readily accessible. As alternative settings continue to offer PEP, monitoring of differences in effectiveness and adherence between two‐ and three‐drug regimens will be essential.

The guidelines also emphasize viewing PEP as an educable moment. Some PEP‐eligible individuals may be at continued risk for HIV infection during subsequent exposures. The PEP encounter can be used to facilitate a transition to PrEP [54]. Conversely, the WHO guidelines also recommend a PrEP to PEP transition. Because such a transition can be nuanced based on the number of PrEP doses taken in the 7 days prior to the exposure, the formulation of PrEP being used and the type of exposure [55, 56, 57, 58], the WHO provides a table to guide providers. Additionally, should PEP be indicated for an individual with a prescription for a two‐drug PrEP regimen, two‐drug PEP should be started immediately, and transitioned to a three‐drug regimen as soon as possible. For individuals already using long‐acting PrEP regimens, such as injectable cabotegravir (CAB‐LA) [59], the extended half‐life means that if someone is not adherent or discontinues the medication, sub‐inhibitory concentrations will persist for weeks or even months, possibly selecting for drug‐resistant HIV strains if the patient is subsequently exposed during the tail period. To protect against the selection for resistance, the WHO recommends PEP be discussed with and available to people who have decided to stop CAB‐LA.

The 2024 WHO guidelines recommend that the full 28‐day course of PEP be dispensed when individuals present after a high‐risk exposure. Further, the guidelines permit the prescription of a full 28‐day course of PEP in case of subsequent exposure. Another approach to “PEP in pocket” not included in the 2024 guidelines is the use of “starter packs” or a few days’ worth of medication prescribed by a clinician to facilitate PEP initiation as soon as possible after an exposure [60]. The use of starter packs was found to be acceptable with excellent follow‐up, and may reduce the time between exposure and initiation of antiretroviral prophylaxis [61]; starter packs may be particularly useful for individuals with occasional unanticipated risk (e.g. not able to plan to use on‐demand PrEP). The 2024 guidelines do not recommend starter packs, citing observational evidence that adherence is increased if a full 28 days’ worth of medication is given at once [62]. Such observational data, however, may need to be re‐considered as more data emerge from community‐based delivery models, which may enhance adherence and access to additional medication among those who are provided starter packs.

### 2.3 Considerations for implementing the new guidelines

While the overall trajectory of the new PEP guidelines continues to emphasize increasing accessibility of PEP, there are several important considerations for implementing the new guidelines. First, to facilitate the benefits of community‐based PEP, access to medications still must be assured. To optimize adherence to the 28‐day course of PEP, providers should be trained to provide adherence support, similar to counselling provided for PrEP initiation [63]. Similarly, task‐sharing requires training of the new workforce in all aspects of PEP delivery, from identification of eligible individuals, to regimen selection, monitoring and follow‐up (including appropriate transition to and from PrEP).

Yet, each context in which PEP is implemented will require a tailored approach and templates should be developed to facilitate PEP delivery in each setting. For example, pharmacy‐delivered PEP programmes and programmes based on community health workers will require different approaches than clinic and health‐post‐based programmes. Templates that outline how PEP should be offered, the type of adherence support that should be utilized, and in which instances referrals are necessary might help to tailor such delivery programmes.

The facilitators and barriers to PEP uptake also differ by patient population. PEP uptake among survivors of sexual assault may be limited by trauma‐specific factors [26, 30, 49, 64, 65], and providers should be trained to offer first‐line support and to refer for additional services—as emphasized in the new WHO guidelines. Such care will be notably different from PEP delivered to a healthcare worker after an occupational exposure. Similarly, uptake among cisgender men who have sex with men may differ from transgender women, analogous to what has been seen in the PrEP continuum among these two populations [66], and disaggregating sex from gender will be an important component of future work delineating barriers to and facilitators of PEP uptake. Therefore, templates should also be developed to support the various settings in caring for the heterogeneous populations among whom PEP may be warranted.

Given the narrow window of time between exposure and when PEP is no longer efficacious, efforts to increase community awareness may synergize with the expansion of settings and provider types that can offer PEP. Increased awareness in the community can decrease the time to presentation after an exposure, which may be further reduced by the decentralization of PEP delivery under the new guidelines. Increased community awareness may also reduce stigma around PEP and HIV [67], further augmenting PEP uptake.

Finally, PEP is not an intervention used in isolation. Increased serostatus awareness through increased HIV testing will facilitate an individual's ability to appropriately assess their need for PEP, and to implement other prevention methods (e.g. condoms, PrEP, etc.). Additionally, PEP implementation will likely be ineffective if structural barriers are not overcome. Antiretroviral deserts—areas where no antiretroviral medications are available—must be overcome via systematic training of providers, establishing delivery centres, and assurance of medication supply [15]. Policies that persecute same‐sex relationships [17], particularly in areas with a high prevalence of HIV infection—areas where PEP may be most needed and effective—must be ended in order to facilitate PEP delivery.

### 2.4 Emerging PEP strategies

For PrEP, public health officials use the PrEP‐to‐need ratio [68] to track uptake among those with an indication for its use. However, similar heuristics do not exist for PEP. Without such metrics, our ability to adapt PEP delivery programmes to areas most in need remains limited. Similarly, without a mechanism by which to track PEP use among eligible individuals, evaluating the impact of expanded PEP access will remain challenging. Such metrics would need to include the proportion of individuals with indications for PEP, PEP prescribing practices and PEP acceptance as well as adherence.

Novel formulations of and delivery mechanisms for antiretroviral therapy and PrEP are also being developed that have the potential to be leveraged for PEP, facilitating further decentralization. Alternative delivery options such as topical gels, vaginal inserts and monthly oral pills are under investigation [69]. Such strategies may help to overcome some acceptability and adherence barriers, particularly for people who are opposed to taking daily tablets. Long‐acting subdermal implants have shown promising early phase results, and will soon undergo PrEP efficacy trials [70, 71]. However, whether or not such agents can be initiated as an immediate PEP‐to‐PrEP transition among individuals with ongoing risk for HIV acquisition remains to be studied.

Long‐acting oral and injectable therapy also holds promise for simplified PEP regimens. Two phase 1 trials of long‐acting oral islatravir demonstrated it was safe and tolerable when used for treatment [72, 73]. However, in light of reports of lymphopenia associated with islatravir [74], it will likely not be utilized as prophylaxis, although the treatment dose was higher than the dose used for PrEP. Every six month injections of lenacapravir were also recently shown to be safe and effective in preventing HIV acquisition among men who have sex with men as well as among transgender and non‐binary individuals [75]. MK‐8527 is a long‐acting nucleoside reverse transcriptase translocation inhibitor, which was recently shown to be well tolerated in both single‐ and multi‐dose forms [76]. A clinical trial is ongoing evaluating a single monthly dose of MK‐8527 in humans (NCT05494736). Several studies have demonstrated the efficacy of CAB‐LA for HIV PrEP [77, 78]. Long‐acting lenacapavir is another emerging injectable alternative [79], with recent data demonstrating its superiority to oral PrEP in cisgender African women [80]. And finally, broadly neutralizing antibodies (bNAbs) have shown promising pre‐clinical data; bNAbs used as PEP were effective in preventing simian‐human immunodeficiency virus when given within 48 hours of an exposure among infant macaque model, and demonstrated half‐lives of several weeks [81, 82].

Importantly, no long‐acting therapy has been evaluated as PEP in humans. Such trials remain logistically and ethically complex. As HIV acquisition is a high‐consequence, low‐probability event, and effective prevention exists, comparative‐effectiveness trials would require extremely large sample sizes, becoming infeasible, while placebo‐controlled trials would be unethical. Thus, observational studies will be essential. However, even if long‐acting PEP is effective, important concerns may limit its use. Many are concerned about the possibility of selecting for drug‐resistant strains should an exposure occur during the extended tail of long‐acting PEP when subinhibitory drug concentrations may be present. To facilitate long‐acting PEP, patients and providers must be educated about the importance of reducing exposures during the tail period. The use of an HIV testing strategy that incorporates more sensitive assays, such as HIV RNA assays, may also reduce the risk for resistance selection by facilitating earlier detection and treatment with multi‐drug regimens.

## 3 CONCLUSIONS

PEP remains an important yet underutilized HIV prevention tool. Updated guidelines from the WHO continue the trend of increasing accessibility via strong recommendations for community‐based delivery and provision via task sharing with non‐healthcare professionals. Additional work is needed to develop implementation strategies around those recommendations, while novel formulations of and delivery mechanisms for antiretroviral therapy continue to emerge.

## COMPETING INTERESTS

L‐TA‐B has no conflicts to disclose. KHM has received unrestricted research funding to study postexposure prophylaxis from Gilead Sciences and Merck, Inc. KHM was the co‐chair of the WHO HIV PEP Guidelines Development Group meeting (8–10 November 2023), to which L‐TA‐B was an invited observer.

## AUTHORS’ CONTRIBUTIONS

L‐TA‐B supported the conceptualization of the work, conducted the literature review and drafted the first version of the manuscript.

KHM supported the conceptualization of the work, assisted in the literature review and contributed to revisions of the manuscript.

## FUNDING

The work is partially supported by the Harvard Center for AIDS Research (NIAID P30AI060354, Rajesh Gandhi, PI; Kenneth H. Mayer, Core Director), as well as the National Institute of Allergy and Infectious Diseases (K23AI182453, PI Lao‐Tzu Allan‐Blitz).

## Data Availability

Data sharing is not applicable to this article as no new data were created or analysed in this study.
