# Correction to “Updated guidelines on HIV post‐exposure prophylaxis: continued efforts towards increased accessibility”

**DOI:** 10.1002/jia2.26409

**Published:** 2025-01-08

Allan‐Blitz, L.‐T. and Mayer, K.H. (2024), Updated guidelines on HIV post‐exposure prophylaxis: continued efforts towards increased accessibility. J Int AIDS Soc., 27: e26393. https://doi.org/10.1002/jia2.26393

In the article, the errors detailed below were identified:

In Discussion, 2.4 Emerging PEP strategies, third paragraph, the sentences originally read:

Every six month injections of lenacapravir were also recently shown to be safe and effective in preventing HIV acquisition among men who have sex with men as well as among transgender and non‐binary individuals [75]. MK‐8527 is a long‐acting nucleoside reverse transcriptase translocation inhibitor, which was recently shown to be well tolerated in both single‐ and multidose forms [76]. A clinical trial is ongoing evaluating a single monthly dose of MK‐8527 in humans (NCT05494736). Several studies have demonstrated the efficacy of CAB‐LA for HIV PrEP [77, 78]. Long‐acting lenacapavir is another emerging injectable alternative [79], with recent data demonstrating its superiority to oral PrEP in cisgender African women [80].

The sentences should read:

MK‐8527 is a long‐acting nucleoside reverse transcriptase translocation inhibitor (similar to islatravir), which was recently shown to be well tolerated in both single‐ and multidose forms [75]. A clinical trial is ongoing evaluating a single monthly dose of MK‐8527 in humans (NCT05494736). Several studies have demonstrated the efficacy of CAB‐LA for HIV PrEP [76, 77]. Long‐acting lenacapavir is another emerging injectable alternative [78], with recent data demonstrating its superiority to oral PrEP in cisgender African women [79]. Every six month injections of lenacapravir were also recently shown to be safe and effective in preventing HIV acquisition among men who have sex with men as well as among transgender and non‐binary individuals [80].

In References, nos. 75−82 were reordered and should be:

75. Gillespie G, Carstens RP, Zang X, Vargo R, Karpoor Y, Bhattacharyya A, et al. Safety and pharmacokinetics of MK‐8527, a novel nRTTI, in adults without HIV. Presented at the Conference on Retroviruses and Opportunistic Infections, March 3–6, 2024. Abstract. 2024. Available at: https://www.croiconference.org/abstract/safety‐and‐pharmacokinetics‐ofmk‐8527‐a‐novel‐nrtti‐in‐adults‐without‐hiv. Accessed July 16, 2024.

76. Fonner VA, Ridgeway K, Van Der Straten A, Lorenzetti L, Dinh N, Rodolph M, et al. Safety and efficacy of long‐acting injectable cabotegravir as preexposure prophylaxis to prevent HIV acquisition. AIDS. 2023;37(6):957–66.

77. Delany‐Moretlwe S, Hughes JP, Bock P, Ouma SG, Hunidzarira P, Kalonji D, et al. Cabotegravir for the prevention of HIV‐1 in women: results from HPTN 084, a phase 3, randomised clinical trial. Lancet. 2022;399(10337):1779–89. https://doi.org/10.1016/S0140‐6736(22)00538‐4

78. Bekerman E, Yant SR, Vanderveen L, Hansen D, Lu B, Rowe W, et al. Long‐acting lenacapavir acts as an effective preexposure prophylaxis in a rectal SHIV challenge macaque model. J Clin Invest. 2023;133(16):e167818.

79. Bekker L‐G, Das M, Abdool Karim Q, Ahmed K, Batting J, Brumskine W, et al. Twice‐yearly lenacapavir or daily F/TAF for HIV prevention in cisgender women. N Engl J Med. 2024.

80. Acevedo‐Quinones M, Agwu AL, Avihingsanon A, Benson P, Blumenthal J, Brinson C, et al. Twice‐yearly lenacapavir for HIV prevention in cisgender gay men, transgender, and gender‐diverse people: interim analysis result from the PURPOSE 2 study. Presented at the HIV Research for Prevention Conference, October 6–10, 2024.

81. Shapiro MB, Cheever T, Malherbe DC, Pandey S, Reed J, Yang ES, et al. Single‐dose bNAb cocktail or abbreviated ART post‐exposure regimens achieve tight SHIV control without adaptive immunity. Nat Commun. 2020;11(1):70.

82. Hessell AJ, Jaworski JP, Epson E, Matsuda K, Pandey S, Kahl C, et al. Early short‐term treatment with neutralizing human monoclonal antibodies halts SHIV infection in infant macaques. Nat Med. 2016;22(4):362–68.

The online version of the article has been corrected.

We apologize for these errors.

